# Elevated level of acetylation of APE1 in tumor cells modulates DNA damage repair

**DOI:** 10.18632/oncotarget.12113

**Published:** 2016-09-19

**Authors:** Shiladitya Sengupta, Anil K. Mantha, Heyu Song, Shrabasti Roychoudhury, Somsubhra Nath, Sutapa Ray, Kishor K. Bhakat

**Affiliations:** ^1^ Department of Biochemistry & Molecular Biology, University of Texas Medical Branch, Galveston, TX 77555, USA; ^2^ Department of Genetics, Cell Biology and Anatomy, University of Nebraska Medical Center, Omaha, NE 68198, USA; ^3^ Department of Pediatrics, Hematology/Oncology Division, University of Nebraska Medical Center, Omaha, NE 68198, USA; ^4^ Department of Radiation Oncology, Houston Methodist Research Institute, Houston, TX 77030, USA; ^5^ Center for Animal Sciences, School of Basic and Applied Sciences, Central University of Punjab, Bathinda 151001, Punjab, India; ^6^ Molecular Biology Research & Diagnostic Laboratory, Saroj Gupta Cancer Centre & Research Institute, Kolkata 700063, India

**Keywords:** apurinic/apyrimidinic endonuclease 1 (APE1), BER, acetylation, DNA damage repair

## Abstract

Apurinic/apyrimidinic (AP) sites are frequently generated in the genome by spontaneous depurination/depyrimidination or after removal of oxidized/modified bases by DNA glycosylases during the base excision repair (BER) pathway. Unrepaired AP sites are mutagenic and block DNA replication and transcription. The primary enzyme to repair AP sites in mammalian cells is AP endonuclease (APE1), which plays a key role in this repair pathway. Although overexpression of APE1 in diverse cancer types and its association with chemotherapeutic resistance are well documented, alteration of posttranslational modification of APE1 and modulation of its functions during tumorigenesis are largely unknown. Here, we show that both classical histone deacetylase HDAC1 and NAD^+^-dependent deacetylase SIRT1 regulate acetylation level of APE1 and acetylation of APE1 enhances its AP-endonuclease activity both *in vitro* and in cells. Modulation of APE1 acetylation level in cells alters AP site repair capacity of the cell extracts *in vitro*. Primary tumor tissues of diverse cancer types have higher level of acetylated APE1 (AcAPE1) compared to adjacent non-tumor tissue and exhibit enhanced AP site repair capacity. Importantly, in the absence of APE1 acetylation, cells accumulate AP sites in the genome and show increased sensitivity to DNA damaging agents. Together, our study demonstrates that elevation of acetylation level of APE1 in tumor could be a novel mechanism by which cells handle the elevated levels of DNA damages in response to genotoxic stress and maintain sustained proliferation.

## INTRODUCTION

Mammalian apurinic/apyrimidinic (AP) endo nuclease 1 (APE1) is a ubiquitous and multifunctional protein. It plays a central role in the repair of AP sites generated either spontaneously or as an intermediate during the repair process of oxidative and drug-induced alkylation damage in the genome via the base excision repair (BER) pathway [1–3]. APE1 cleaves the AP-site to generate 3′-OH group and 5′-deoxyribose-5-phosphate (dRP) termini or removes a 3' blocking group by functioning as a 3′ exonuclease [4]. In addition to its DNA repair function, APE1 functions as a transcriptional coregulator of many genes involved in multiple cellular pathways [5–11]. APE1 was also shown to activate DNA-binding of many stress-inducible transcription factors (including c-Jun, P53 and NF-kB) through its redox effector (Ref-1) function [11–13]. Thus, APE1 has a critical role in linking DNA repair to regulation of diverse signaling pathways.

We discovered earlier that human APE1 is acetylated primarily at Lys 6 and Lys 7 by the histone acetyltransferase p300 both *in vitro* and in cells [14]. Acetylation modulates the transcriptional regulatory function of APE1 in up- and downregulation of diverse genes associated with multidrug resistance, cell-cycle control and apoptosis [5, 9, 15]. Dr. Tell's group in collaboration with us have shown that other Lys residues (Lys 27, 31, 32, 35) in the N-terminal domain of APE1 can also be acetylated upon genotoxic stress and mutation of these Lys residues to Ala alters the DNA damage repair activity of APE1 [16]. APE1 was also found to be ubiquitinated at multiple Lys (Lys 24, 25, 27) residues in the N-terminal domain and ubiquitination at these residues can modulate the stability or localization of APE1 [17, 18]. Other posttranslational modifications such as phosphorylation and nitrosylation have been shown to alter multiple functions of APE1 [18–22]. The disordered and conserved N-terminal domain of APE1 harboring the multiple acetylation sites is the common interaction domain for multiple partners in diverse pathways including transcriptional regulation [5, 7–10], and RNA processing [23, 24]. Importantly, we discovered that both the DNA repair function and acetyl-acceptor Lys 6 and 7 sites in APE1 are essential for cell proliferation and survival [25]. Similarly, other BER proteins, including NEIL2 and OGG1 have also been found to be acetylated, modulating their DNA repair function [26, 27].

Overexpression of APE1 in cancer cell lines and tumour tissues from various sources including non-small cell lung cancer (NSCLC), colon, glioma, head and neck, breast, and its association with resistance to various anticancer drugs strongly establishes APE1 as a target for cancer therapy [28–36]. However, little is known about alteration of posttranslational modifications of APE1 during tumorigenesis. Recently, we have shown that the N-terminal domain (1-33 amino acids; aa) of APE1 is cleaved by a limited proteolysis in tumor, acetylation of multiple Lys residues in this domain prevents this proteolysis [37].

Here, we examined the regulation of acetylation of APE1 in cells by the interplay of both classical and NAD^+^-dependent histone deacetylases. We found that acetylation increases the DNA repair activity of APE1, and absence of this acetylation contributes to accumulation of AP sites in the genome and increased cell sensitivity towards both alkylating and oxidative agents. Primary tumor tissues of various cancer types have elevated levels of AcAPE1 and exhibit significantly enhanced AP site repair capacity. Together, our study suggest that increased levels of AcAPE1 in tumor plays a critical role in their survival and sustained proliferation in response to genotoxic stress.

## RESULTS

### Elevated levels of AcAPE1 in tumor tissue

**Figure 1:**
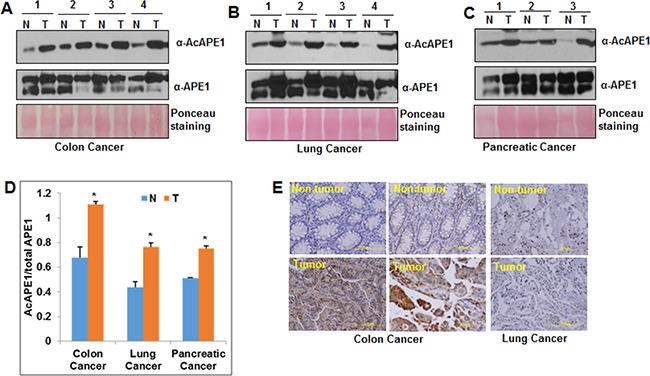
Elevated levels of AcAPE1 in tumor tissue **A, B** & **C.** Western blot analysis of tumor-adjacent non-tumor (N) and tumor (T) tissue lysates from colon (A), lung (B) and pancreatic (C) cancer patients with α-AcAPE1, APE1 Abs; ponceau staining used as loading control. **D.** The levels of APE1 and AcAPE1 in these tissue samples were quantitated using ImageJ and the ratio of AcAPE1/total APE1 was represented in a histogram with mean ± SD; p value < 0.05 shown as * (Student T Test). **E.** Representative immunohistochemical staining of AcAPE1 in paraffin-embedded colon and lung cancer tissue sections.

We compared AcAPE1 level in primary tumor tissues to adjacent non-tumor (normal) tissues from patients with colon, non-small cell lung cancer (NSCLC) or pancreatic cancer by Western blot analysis (Figure 1A, 1B & 1C) using our previously generated AcAPE1-specific antibody [5]. We have previously shown that this antibody is highly specific in recognizing AcAPE1 species (acetylated at Lys 6 position) and does not cross react with 50-fold excess of unmodified APE1 [5]. We found that the fraction of APE1 present in acetylated form (AcAPE1/total APE1) was significantly higher in tumor tissues as compared to adjacent non-tumor tissues (Figure 1D and Supplementary Figure S1A, S1B & S1C). Immunohistochemical analysis also confirmed increased nuclear AcAPE1 staining in tumor compared to non-tumor tissues (Figure 1E). These data indicate that tumor tissues of diverse cancer types have elevated levels of AcAPE1 as compared to the adjacent non-tumor tissues.

### Both classical histone deacetylase HDAC1 and NAD^+^-dependent deacetylase SIRT1 are involved in deacetylation of APE1 in cells

**Figure 2:**
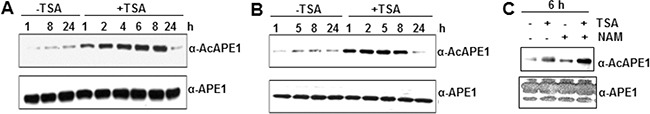
Effect of classical HDAC inhibitor TSA and non-classical deacetylase inhibitor NAM on APE1 acetylation in cells **A** & **B.** HCT116 (A) and hTERT-BJ (B) cells were treated with TSA (100 ng/ml) and the levels of AcAPE1 (upper panel) or APE1 (lower panel) were measured at the indicated time points by Western blot analysis. **C.** HCT116 cells were treated with either 100 ng/ml TSA or 10 mM NAM or both for 6 hrs and the level of AcAPE1 (upper panel) or APE1 (lower panel) were measured by Western blot analysis.

Deregulation of the fine balance between histone acetyl transferases and deacetylases in tumor cells may affect the acetylation level of APE1 during tumorigenesis. We showed previously that p300 is the major acetyltransferase for acetylation of APE1 at Lys 6 and 7 residues in cells [14]. To ask whether classical histone deacetylases (HDAC1-11) are also involved in deacetylation of AcAPE1, we treated colon adenocarcinoma HCT116 cells with trichostatin A (TSA), a specific inhibitor for classical HDACs [38], and measured AcAPE1 level. We found that TSA treatment significantly increased the level of AcAPE1 (> 5-fold) after as little as 1 hour (h) of treatment (Figure 2A, upper panel; Supplementary Figure S2A), without changing the total level of APE1 (Figure 2A, lower panel). AcAPE1 returned to the normal level by 24 h. Similarly, TSA treatment increased AcAPE1 levels in hTERT-transformed human primary skin fibroblast BJ cells (Figure 2B; Supplementary Figure S2B). These data indicate that classical deacetylases are involved in regulating acetylation of APE1 in cells. Because NAD^+^-dependent non-classical histone deacetylase SIRT1 was also shown to be involved in deacetylation of AcAPE1 [39], we found that treatment with NAD^+^-dependent deacetylase inhibitor nicotinamide (NAM) increased the AcAPE1 level, while combined treatment of both NAM and classical deacetylase inhibitor TSA had an additive effect (Figure 2C; Supplementary Figure S2C). Thus both classical and NAD^+^-dependent HDACs along with p300 [14] are involved in maintaining the homeostasis of the acetylation/deacetylation cycles of APE1 in cells.

**Figure 3:**
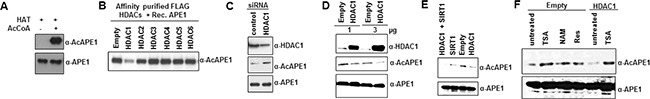
HDAC1 and SIRT1 are responsible for APE1 deacetylation **A.** Recombinant APE1 (2 μg) was incubated with p300 HAT domain ± 1mM AcCoA for 2 h at 30°C and then 50 ng of unmodified or acetylated APE1 was used for Western blot analysis with Abs specific for AcAPE1 or APE1. **B.** Recombinant *in vitro* acetylated APE1 (200 ng) was incubated with immunoprecipitated FLAG-HDACs (eluted with FLAG peptide) from FLAG-HDAC1-6 transfected HEK293T cells. The levels of AcAPE1 were measured by Western blot analysis. **C.** HEK293T cells were transfected with 80 nM of HDAC1 specific or control duplex siRNA. HDAC1, AcAPE1 and APE1 levels in cell extracts were measured 48 h after transfection by Western blot analysis. **D.** HEK293T cells were transfected with 1 or 3 μg of HDAC1 expression plasmid. Forty eight hours after transfection the AcAPE1 and APE1 levels were measured in the cell extracts by Western blot analysis. **E.** HEK293T cells were transfected with either SIRT1 or HDAC1 or both expression plasmids and then the level of AcAPE1 and APE1 were measured by Western blot analysis. **F.** HEK293T cells were transfected with either empty vector or HDAC1 expression plasmids and then treated with TSA, NAM or resveratrol as indicated and the levels of AcAPE1 and APE1 were measured by Western blot analysis.

Our earlier studies showed that APE1 is associated with Class I (HDAC1-3) but not with Class II (HDAC 4-6) deacetylases [14]. We asked which HDAC deacetylates AcAPE1 *in vitro*. At first, we generated AcAPE1 *in vitro* by incubating purified recombinant wild type (WT) APE1 [5] with p300 HAT domain either in the presence or absence of acetyl coenzyme A (AcCoA), and confirmed acetylation by Western blot analysis using our AcAPE1 specific antibody (Figure 3A). Next, we incubated *in vitro* AcAPE1 with affinity-purified FLAG-HDACs (from HCT116 cells ectopically expressing these individual HDACs), followed by examination of AcAPE1 level by Western blot analysis. We found that only HDAC1 but not HDAC2-6 can efficiently catalyze deacetylation of AcAPE1 *in vitro* (Figure 3B; Supplementary Figure S3A). To further confirm that HDAC1 is involved in regulating the acetylation level of APE1 in cells, we downregulated endogenous HDAC1 level using HDAC1-specific siRNA. Figure 3C and Supplementary Figure S3B show that downregulation of HDAC1 level significantly increased APE1 acetylation. Consistent with this, ectopic expression of HDAC1 reduced AcAPE1 level (Figure 3D & 3E; Supplementary Figure S3C, S3D & S3E), and combined ectopic expression of HDAC1 and SIRT1 reduced AcAPE1 level to a greater extent (Figure 3E; Supplementary Figure S3E). Moreover, the effect of ectopic HDAC1 expression on AcAPE1 level was overcome in the presence of deacetylase inhibitor TSA (Figure 3F; Supplementary Figure S3F). Although resveratrol was shown to activate SIRT1 [40], treatment with resveratrol did not decrease AcAPE1 level (Figure 3F). Together, these data indicate that both classical HDAC1 and NAD^+^-dependent HDAC are involved in maintaining the homeostasis of acetylation/deacetylation cycles of APE1 in cells.

### Modulation of acetylation level of APE1 in cells affects AP site cleavage activity *in vitro*

**Figure 4:**
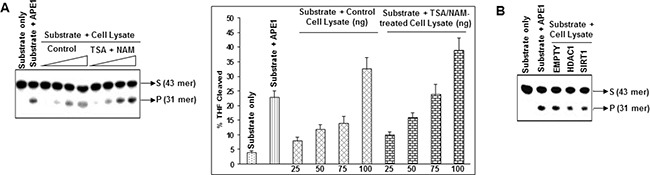
Modulation of AcAPE1 levels in cells alters AP site repair capacity of their extracts **A.** HCT116 cell were treated with or without TSA (100 ng/ml) and NAM (10 mM) for 6 hrs. Various amount (25-100 ng) of cell extracts was incubated with 5'^32^p-labeled THF (reduced AP site) containing 43-mer duplex oligonucleotide (substrate: S) at 37°C for 3 min, and the cleaved product (P) was separated as described in Materials & Methods; side panel: the percentage of cleaved product from the substrate was quantitated and plotted with ± SD from 2-3 independent experiments. **B.** HCT116 cells were transfected with empty vector or HDAC1 or SIRT1 expression plasmids and 48 h after transfection, 100 ng cell extracts from each sample were used for AP-endonuclease activity assay as described in (A).

To explore whether modulation of APE1 acetylation level in cells affects its DNA repair activity, we treated HCT116 cells with TSA and NAM for 6 h and then measured the AP-endonuclease activity in cell extracts using THF containing oligonucleotide substrate as described before [41]. A dose-dependent increase in the THF cleavage activity of cell extracts was observed after treatment with TSA and NAM (Figure 4A). Because treatment with TSA and NAM enhanced AcAPE1 level without changing the total APE1 (Figure 2C), these results indicate that the increased AP site cleavage activity of TSA and NAM treated cell extracts is due to enhanced APE1 acetylation. Furthermore, we ectopically expressed HDAC1 and SIRT1 (which are primarily involved in deacetylation of APE1; Figure 3; [39]), and measured the AP-endonuclease activity of the cell extracts. Ectopic expression of either HDAC1 or SIRT1 decreased the THF cleavage activity of the cell extracts (Figure 4B; Supplementary Figure S4). Thus modulation of acetylation levels of APE1 in cells plays a key regulatory role in AP site repair capacity.

### Acetylation of APE1 enhances its endonuclease activity

**Figure 5:**
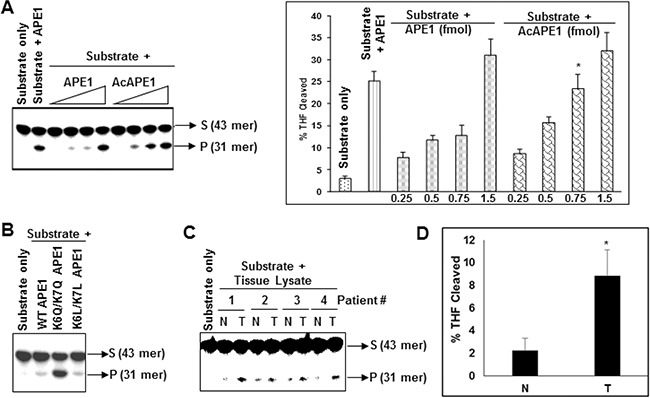
Acetylation of APE1 enhances its AP-endonuclease activity and tumor tissues have higher AP-endonuclease activity **A.** Incision of the 5'^32^p-labeled THF (reduced AP site)-containing 43-mer duplex oligonucleotide by 0.1, 0.5, 1.0 or 5.0 fmol of recombinant APE1 or AcAPE1; side panel: quantitation of the percentage of cleaved product from the substrate shown. **B.** Comparison of AP-endonuclease activity of WT, acetylation mimic K6Q/K7Q (QQ) or K6L/K7L (LL) mutant APE1 proteins. **C.** Tissues extracts containing equal amount of APE1 from non-tumor (N) or tumor (T) tissues of lung cancer patients were incubated with 5'^32^p-labeled THF-containing duplex oligo and the cleaved product was separated as described before. **D.** Quantitation of the percentage of THF cleaved product in the non-tumor and tumor samples from C and plotted with ±SD.

The observation that modulation of AcAPE1 level in cells alters AP site repair capacity of cell extracts *in vitro* (Figure 4), raises the possibility that acetylation of APE1 could modulate its DNA damage repair activity. We investigated directly whether acetylation affects the AP-endonuclease activity of APE1 *in vitro*. We found that acetylation of APE1 increased its AP-endonuclease activity in a dose-dependent manner (Figure 5A). Consistent with this we observed that mutation of two acetylable Lys 6 & 7 to acetylation-mimic Gln (K6Q/K7Q) which eliminates the positive charge and resemble acetyl-Lys, significantly increased APE1's endonuclease activity *in vitro* (Figure 5B; Supplementary Figure S5A). However, to our surprise we observed that mutation of Lys 6 & 7 to neutral aa Leu (K6L/K7L) which eliminates the positive charge but does not resemble acetyl-Lys, could not enhance AP-endonuclease activity. These results suggest that acetylation of APE1 enhances its AP endonuclease activity.

### Tumor tissue extracts have increased AP-site cleavage activity

Consistent with our observation that acetylation significantly increased endonuclease activity of APE1 (Figure 5A & 5B), extracts of NSCLC tissue samples showed significantly enhanced AP site cleavage activity with respect to matched non-tumor control when tissue extracts with equivalent amount of APE1 were used in *in vitro* cleavage assay (Figure 5C & 5D; Supplementary Figure S5B). This suggests that the observed higher AcAPE1 level in tumor tissues contributes to enhanced AP site repair capacity of their extracts.

### Absence of acetylation of APE1 contributes to accumulation of AP sites in the genome

**Figure 6:**
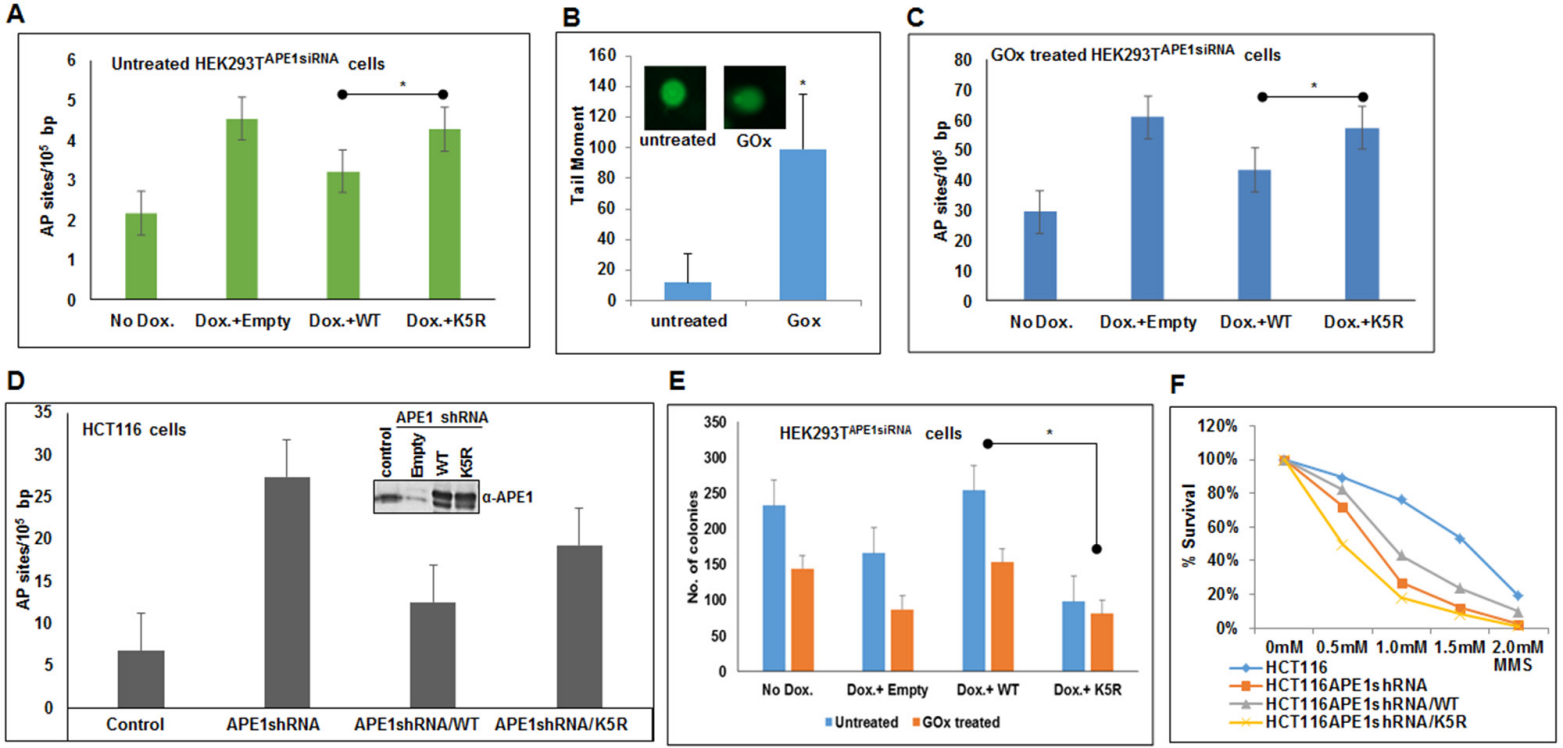
Absence of APE1 acetylation leads to accumulation of AP sites in the genome and sensitizes cells to DNA damaging agents **A** & **C.** Endogenous APE1 was downregulated in HEK293T^APE1siRNA^ cells with Dox treatment. FLAG-tagged WT or acetylation defective (mutations of Lys 6, 7, 27, 31 & 32 to non-acetylable Arg; K5R) mutant APE1 was ectopically expressed in these cells. AP sites in the genomic DNA were measured using ARP kit in glucose oxidase (GOx; 100 ng/ml for 30 mins) treated (C) or untreated control (A) cells. Bar diagram representing number of AP site/10^5^ bp. Error bars indicate mean ±SD (n=3). **B.** FPG Comet assay to assess relative quantitation of oxidative DNA damage after GOx treatment. Histogram showing mean tail moment (50-100 randomly selected cells) after GOx treatment; representative images of Comet slides shown in inset. **D.** Quantitation of AP sites in control, APE1 shRNA expressing HCT116 cells, WT or K5R mutant APE1 expressing HCT116 cells after endogenous APE1 downregulation (expression level shown in inset) as in A & C. **E** & **F.** DNA damage cell sensitivity assay measured by clonogenic assay (E) after GOx treatment in endogenous APE1 downregulated HEK293T^APE1siRNA^ cells (by Dox treatment) and after ectopic expression of WT or K5R mutant APE1in endogenous APE1 downregulated cells, and (F) in APE1 shRNA expressing HCT116 cells transfected with WT or K5R APE1 and treated with different dose of MMS and plotted as % survival. Details are in Materials and Methods. * represents p value < 0.05 (Student T Test).

The observation that acetylation of APE1 enhances its endonuclease activity raises the possibility that in the absence of acetylable Lys residues in APE1, cells will accumulate AP sites in the genome. We quantitated AP sites in the genome of cells expressing WT and non-acetylable K5R (acetylable Lys 6,7,27,31,32 mutated to Arg) mutant by using an aldehyde-reacting probe as described elsewhere [42–44]. We used HEK293T^APE1siRNA^ cells stably expressing APE1 siRNA under doxycycline (Dox)-inducible promoter [9]. We treated the cells with Dox to downregulate endogenous APE1 and then ectopically expressed FLAG-tagged WT APE1, or its mutant lacking the acetylation sites [37]. We observed that depleting endogenous APE1 increased AP sites in the genome compared to control (Figure 6A). Importantly, a significant increase in AP sites was observed in the genome of cells expressing K5R APE1 mutant compared to WT APE1 expressing cells (Figure 6A). Furthermore, we treated these cells with glucose oxidase (GOx) which produces hydrogen peroxide and induces oxidative damages which in turn produces AP sites in the genome [27, 45, 46]. We first confirmed that GOx treatment indeed induced oxidative DNA damage by Fpg-alkaline Comet assay. As shown in Figure 6B, treatment with GOx enhanced significant DNA breaks in the genome as evidence by increased comet tail in GOx-treated cells as compared to control. Consistent with this, treatment with GOx significantly enhanced AP sites in the genome of APE1 downregulated cells which can be relieved by ectopic expression of WT APE1 but not by the nonacetylable K5R mutant (Figure 6C). This indicates that APE1 acetylation plays an important role in DNA damage or AP site repair in cells. Furthermore, we also quantitated AP sites in control and APE1-downregulated HCT116 cells that stably express APE1-specific shRNA. As expected, we found that downregulation of endogenous APE1 significantly accumulated AP sites in the genome which was rescued by ectopic expression of WT APE1 but not by the K5R mutant APE1 (Figure 6D).

### APE1 acetylation plays a role in cell survival in response to genotoxic stress

We examined the role of APE1 acetylation in cell survival and/or proliferation. As expected, we observed that depleting endogenous APE1 with Dox significantly decreased the number of viable HEK293T^APE1siRNA^ colonies compared to control (Figure 6E). This effect can be rescued by ectopic expression of WT APE1, but not with non-acetylable K5R mutant APE1 (Figure 6E). We further examined the role of APE1 acetylation in cell proliferation after induction of DNA damage. Treatment with GOx decreased the number of viable colonies which can be rescued by ectopic expression of WT APE1, but not with its non-acetylable mutant (Figure 6E). Furthermore, we found that treatment with alkylating agent methylmethane sulphonate (MMS) sensitized HCT116 cells that express APE1-specific shRNA (Figure 6F). While ectopic expression of WT APE1 protected these cells to some extent, expression of non-acetylable K5R mutant sensitized the cells to MMS (Figure 6F). These data indicate that APE1 acetylation plays a critical role in cell survival and/or proliferation and that the absence of APE1 acetylation sensitizes cells to DNA damaging agents.

## DISCUSSION

Despite the fact that the N-terminal acetylable Lys residues in APE1 are dispensable for its DNA repair activity *in vitro* [47], this study unraveled the novel role of acetylation of APE1 in AP site repair both *in vitro* and in cells. Furthermore, our study provides evidence that alteration of acetylation level of APE1 in diverse primary tumor tissues plays a critical role for cell survival and proliferation. AP sites in the genome are generated spontaneously or after excision of the damaged bases by DNA glycosylases during BER [2, 3, 48]. Approximately, 10,000 such lesions are continuously generated endogenously in the genome/cell/day [49]. Unrepaired AP sites block DNA replication and transcription and are highly susceptible to spontaneous cleavage and thus DNA strand breaks, leading to cell apoptosis [50–54]. Thus the DNA repair function of APE1 plays an essential role to protect cells from both endogenous and exogenous DNA damage. Our current study shows that acetylation enhances its AP-endonuclease activity and plays an essential role in cell proliferation and survival in response to DNA damages.

Tumor cells are proliferating, transcriptionally very active and have energy demands, which produce oxidative stress [55, 56]. All these factors contribute to enhanced generation of AP sites in the genome of tumor cell [57–60]. Presence of oxidative damage and AP site clusters in replicating or transcriptionally active unfolded chromatin regions may be prevalent in tumor. Thus, there is urgency to repair AP sites faster and efficiently in tumor cells for their survival and proliferation. The AP-endonuclease activity of APE1 should be enhanced in order to repair increased AP sites in tumor cells, which can be achieved by enhancing its acetylation. Consistent with this idea, our data show that primary tumor tissue of diverse origins has higher acetylated APE1 compared to non-tumor tissue. Thus, our study suggests that alteration of posttranslational modification (i.e. acetylation) of APE1 could be a novel mechanism that cancer cells exploit to handle the elevated levels of DNA damage and maintain sustained proliferation. Enhanced APE1 acetylation after treatment with genotoxic agents such as MMS or GOx [5, 9, 16, 39] supports this scenario. Moreover, given our and other's findings that APE1 acetylation modulates expression of many genes [5, 6, 9, 14, 37], and it enhances its endonuclease activity (present study), it appears that increased APE1 acetylation in tumor cells modulates DNA damage repair, and at the same time also alters gene expression to maintain sustained proliferation.

Although earlier studies showed that the N-terminal domain (1-63 aa) of APE1 is not essential for its AP-endonuclease activity *in vitro* [47], one recent study suggests that neutralization of many charged Lys residues in this domain enhances its DNA repair activity [16]. However, whether posttranslational modification of these Lys residues modulates this function is largely unknown. In this study we show directly that covalent modification of these Lys residues by acetylation modulates DNA repair activity of APE1 both *in vitro* and in cells. Stimulation of APE1's AP-endonuclease activity due to acetylation suggests two possible mechanisms: acetylation in APE1 could either increase its affinity for the substrate AP site in DNA, or decrease APE1's affinity for the product (cleaved AP-site), thus increasing its turnover. Because APE1 remains bound to its product, and that the rate-limiting step in AP site excision is dissociation of APE1 from the product [61–63], it is likely that acetylation enhances APE1's turnover by weakening its interaction with the AP site product. Because acetylation is one of the posttranslational modifications by which the overall positive charge of a protein is neutralized, and Lys 6, 7, 27, 31 and 32 in APE1 can be modified by acetylation, we propose that acetylation of APE1 likely decreases highly positive “charge patch” in its N-terminal domain. This in turn weakens or abrogates the charge-dependent contacts of the N-terminal domain of APE1 with the product cleaved AP site. Interestingly, mutation of the acetylable Lys 6 & 7 to neutral Leu, which eliminates the positive charge but does not resemble acetyl Lys have no significant effect on its AP-endonuclease activity. This also raises the possibility that acetylation not only neutralizes the positive charges in the N-terminal but also puts acetyl marks which may induce conformational changes in APE1 that facilitates substrate binding or release from the product cleaved AP sites. Further studies are necessary to address this issue.

Posttranslational modifications have emerged as the main mechanisms for controlling multiple functions of a protein. Like acetylation, other posttranslational modifications such as phosphorylation, ubiquitination and nitrosylation of APE1 were reported earlier. An earlier study by Yacoub *et al*. [64] showed that APE1 can be phosphorylated *in vitro* by casein kinase I and II (CKI and CKII). CKII-mediated phosphorylation of APE1 abolished DNA repair activity *in vitro*, while phosphorylation by PKC was shown to alter the redox function of APE1 [64, 65]. A subsequent study by Fritz and Kaina showed that APE1 phosphorylation by CKII enhances redox activation of the AP-1 transcription factor and has no effect on its DNA repair activity [66]. Although these studies reported APE1 phosphorylation by CK II *in vitro* and PKC in cells, the phosphorylation sites have not been identified. However, one study has shown that APE1 can also be phosphorylated in human cells at Thr233 in the C-terminal domain by Cdk5 and that phosphorylation reduced the AP-endonuclease activity both *in vitro* and in cells [22]. However, we found that mutation of Thr 233 to phosphomimic Glu had no effect on APE1's acetylation compared to the WT protein (data not shown). Ubiquitination of APE1 at multiple Lys (Lys 24, 25, 27) residues in the N-terminal domain was shown to modulate the stability or localization of APE1 [17–19]. Because acetylation and ubiquitination occurs in the same Lys residues in many proteins and regulate their stability, we examined whether acetylation of APE1 affects its half-life. Surprisingly, we found that different doses of protein translation inhibitor cycloheximide had not much effect on endogenous APE1 as well as ectopic APE1 protein level (Supplementary Figure S6). Moreover, we found that, mutation of the acetylable Lys residue to Arg in APE1 had no significant effect on the stability of APE1 (Supplementary Figure S6C & S6D). We do not know the reason why treatment of cycloheximide showed no effect on APE1 level. Other method such as metabolic labeling of APE1 with S^35^ methionine may enable to resolve this issue. *S*-nitrosylation of APE1, another post-translational modification, has been shown to occur *in vivo.* Treatment with *S*-nitroglutathione, an *S*-nitrosylating agent, stimulated nuclear export of APE1 through *S*-nitrosylation at Cys93 and Cys310 in a CRM1-independent manner [20]. Thus it is evident that the activity of APE1 or its localization could be finely tuned via different post-translational modifications, including phosphorylation, acetylation, nitrosylation, ubiquitination, *etc*., in order to coordinate specific biological functions.

Over the last 20 years, overexpression of APE1 and alteration of its subcellular localization in many cancer types have been established. However, our study provides strong evidence that primary tumor of various cancer types have elevated levels of AcAPE1. The mechanism behind increase in APE1 acetylation level in tumor is currently not known. Because p300 primarily acetylates APE1 in cells [14], and both classical histone deacetylase HDAC1 and NAD^+^-dependent deacetylase SIRT1 are involved in APE1 deacetylation in cells [14, 39], deregulation of the fine balance between acetyl transferase p300 and HDAC1/SIRT1 deacetylase activity in tumor may elevate APE1 acetylation during tumorigenesis. However, our results on p300, HDAC1 and SIRT levels in non-tumor vs. tumor tissues (data not shown) could not explain enhanced APE1 acetylation in tumor cells. We have recently shown that acetylation level of APE1 increases in S-phase of the cell cycle [37], when p300 is also activated [67], and thus cell proliferation could be a trigger for enhanced APE1 acetylation in cancer Furthermore, enhanced phosphorylation of APE1 by PKC or Cdk5 which is often overexpressed in some cancers may alter the acetylation level of APE1 [22, 68]. Several studies have shown that multiple signal-dependent modifications (phosphorylation, acetylation, etc.) of a protein occur sequentially, with one modification influencing the subsequent ones [69]. Because APE1 is known to be phosphorylated [65], it is possible that such modification of APE1 in cancer cells enhances its interaction with p300 to induce acetylation or decrease its interaction with HDAC1/SIRT1 to maintain enhanced level of APE1 acetylation.

In summary, this study shows that APE1 acetylation enhances its AP-endonuclease activity and that enhanced acetylation of APE1 in tumors could contribute in protecting cells from endogenous and drug-induced DNA damages. Because APE1 acetylation can modulate both its transcriptional regulatory functions and DNA damage repair function, our study implicates that the AcAPE1 rather than total APE1 levels in tumor could be used as a predictive marker in cancer. Although extensive studies are being carried out on targeting the DNA repair or redox function of APE1 to sensitize tumor cells [70–74], our study provides a rationale for future development of AcAPE1 targeted therapeutic regimens.

## MATERIALS AND METHODS

### Patients' tissue samples, extraction of tissue lysates and western blot analysis

The resected frozen tissues from non-small cell lung carcinoma (NSCLC), colon and pancreatic cancer patients (both tumor and adjacent non-tumor) were collected from University of Texas Medical Branch (UTMB; Galveston) Cancer Center tissue bank. Tissues were collected in accordance with institution's review board approval. Cell lysates were prepared with approximately 100 mg of tissue, washed in phosphate buffered saline (PBS) pH 7.4. Each tissue was minced into fine pieces and homogenized using a glass dounce homogenizer in 1 ml of cold lysis buffer containing 50 mM Tris-HCl pH 7.5, 150 mM NaCl, 1% Triton X-100, 0.1 mM EDTA and protease inhibitor cocktail buffer tablet (PI; Roche Diagnostics). Lysates were centrifuged at 14,000 rpm for 20 min at 4°C and the supernatants were stored at −80°C. Tissue extracts equivalent to 30-60 μg of total protein were separated by SDS/PAGE, transferred to nitrocellulose membrane for Western blot analysis with 1:2000 dilution of mouse monoclonal Ab (Novus Biologicals; # NB100-116) and 1:300 dilution of rabbit polyclonal acetylated APE1 (AcAPE1) Ab [5]. Bands were quantitated using Image J analysis tool.

### Cell lines, plasmids, siRNAs, transfection and treatments

Human embryonic kidney HEK293T (ATCC # CRL-3216) and doxycycline inducible APE1 downregulated ^APE1siRNA^HEK293T cells were cultured in DMEM-high glucose medium with 10% fetal calf serum (FCS; Sigma) and antibiotic mixture of 100 U/ml penicillin and 100 μg/ml streptomycin (Gibco-BRL) as described previously [9]. Human colon cancer HCT116 (ATCC #CCL-247) cells were grown in MCoy's 5A medium. Generation of HCT116 cells stably expressing APE1 shRNA or control shRNA were described previously [15]. hTERT-immortalized human foreskin fibroblast BJ-5ta (ATCC #CR-4001) cells were cultured in DMEM-low glucose medium (Gibco-BRL) with FCS and antibiotics. All cell lines were authenticated by STR DNA profiling on August, 2015 by Genetica DNA laboratories, Burlington, NC. HCT116 or BJ-hTERT cells were treated with TSA (100 ng/ml; Calbiochem) or nicotinamide (10mM; Sigma) for various time periods and whole cell extracts were prepared for Western blot analysis. Classical histone deacetylase HDAC1-HDAC6 expression plasmids and SIRT1 expression plasmid described earlier [5, 14, 75] were transfected in HK293T cells. HDAC1 level was downregulated by transfecting cells with HDAC1 siRNA (Dharmacon). Mutation of residues Lys 6, 7, 27, 31, 32 to Arg (K5R) in APE1-FLAG-tagged pCMV5.1 plasmid were generated using a site-directed mutagenesis Kit (Stratagene) following manufacturer's protocol. Exponentially growing HCT116 cells stably expressing APE1-shRNA were transfected with FLAG-tagged PCMV5.1 expression plasmid containing wild type (WT) or K5R mutant APE1. ^APE1siRNA^HEK293T cells were treated with doxycycline (Dox; 1 ug/ml) for 5 days to knockdown endogenous APE1 followed by transfection with FLAG-tagged WT APE1 or K5R mutant APE1 as described elsewhere [5, 9]. Cells were transfected using Lipofectamine 2000 following manufacturer's protocol and harvested after 48 h for cell lysate preparation. For Western blot analysis, α-APE1[76], α-AcAPE1, α-HDAC1 (EMD Millipore; # 06-720) Abs were used.

### *In vitro* acetylation/deacetylation assay

Recombinant APE1 was purified as described previously [41], and was incubated with p300 HAT domain ± 1mM AcCoA for 2 h at 30°C and then 50 ng of unmodified or AcAPE1 was used for Western blot analysis with Abs specific for AcAPE1 or APE1 [5, 9]. For *in vitro* deacetylation assay, lysates from HEK293T cells transfected with individual FLAG-tagged HDACs 1-6, were immunoprecipitated with α-FLAG Ab cross-linked to agarose beads (Sigma; A2220) and eluted with FLAG peptide. 200 ng of AcAPE1 was incubated with immunoaffinity purified HDACs at 37°C for 1 h and subjected to Western blot analysis.

### Apurinic/apyrimidinic (AP) endonuclease activity measurement assay

A 43-mer oligonucleotide containing AP site analog tetrahydrofuran (THF) at nucleotide 31 (Midland Corp) prepared as described previously was 5′-end-labeled with [γ-^32^P] ATP using T4 polynucleotide kinase [5, 41, 76]. Following annealing to the complementary strand with A opposite THF, the duplex oligomer was purified by gel filtration column (Chroma Spin TE 10; Clontech). This THF-containing duplex oligomer was incubated with recombinant WT APE1, recombinant AcAPE1, mutant APE1 proteins, or cell lysates as described earlier at 37°C for 3 min during which the reaction rate was linear in a 15 μl reaction mixture containing 50 mM Tris-HCl pH 8.5, 50 mM KCl, 1 mM DTT, 0.1 mM EDTA and 100 μg/mL bovine serum albumin and 2mM MgCl_2_. The reaction was stopped with 10 μl 80% formamide/40 mM NaOH containing 0.05% xylene cyanol, followed by heating at 95°C for 5 min; the samples were kept on ice until ran in denaturing gel electrophoresis in 20% polyacrylamide containing 8 M urea to separate the substrate oligomer from the cleaved product. The gels were dried and the radioactivity was quantitated by phosphoimager analysis in a Storm system (Molecular Dynamics).

### AP site measurement assay

Endogenous APE1 was downregulated in ^APE1siRNA^HEK293T cells with Dox (Sigma; 1 μg/ml) treatment [9]. Then the cells were transfected with WT, or mutant APE1 expression plasmids, as described above. Forty eight hours post transfection, total genomic DNA was isolated by Qiagen Dneasy kit following manufacturer's protocol. AP sites were measured using aldehyde reactive probe (Dodinjo, Japan) according to manufacturer's protocol.

### Comet assay

Trevigen's FPG FLARE (Fragment Length Analysis using Repair Enzymes) Comet assay kit was used following manufacturer's protocol. Briefly, control and GOx treated cells were lysed in Comet slides in low melting agarose followed by incubation with FPG before alkaline electrophoresis. DNA in the nucleoid was visualized by SYBR Gold staining in a fluorescence microscope (EVOS FL auto, Life Technologies). Data analysis was performed using Open Comet of Image J program (NIH) in 50-100 randomly selected cells and plotted as mean Tail moment.

### Clonogenic assay

HCT116 cells stably expressing APE1 shRNA were transfected with WT or mutant APE1 expression plasmid. After forty 48 hours of transfection, approximately 500 cells plated on 60-mm dishes were treated with various doses of methylmethane sulphonate (MMS; Sigma; 0.5, 1, 1.5 and 2mM) for 1 hour, washed in PBS and allowed to grow in fresh medium for two weeks until visible colonies appear. HCT116 cells expressing control shRNA used as a control. The colonies were fixed with 100% methanol, stained with Giemsa staining solution (1:50) and counted. Similarly^APE1siRNA^HEK293T cells were treated with Dox for 5-6 days to knockdown endogenous APE1. Cells were transfected with WT or mutant APE1 expression plasmid. Colony formation assay was performed after treatment GOx as described above.

### Statistical analysis

Statistical analysis was done using Student T Test and p value less than 0.05 was considered significant.

## SUPPLEMENTARY FIGURES


