# Supplementary material for: Elevated level of acetylation of APE1 in tumor cells modulates DNA damage repair
Source: Oncotarget. 2016 Sep 19;7(46):75197–209. doi: 10.18632/oncotarget.12113 (PMC5342734; doi:10.18632/oncotarget.12113)
Supplement: Supplementary file 1 [file oncotarget-07-75197-s001.pdf]

Elevated level of acetylation of APE1 in tumor cells modulates DNA damage repair

SUPPLEMENTARY FIGURES

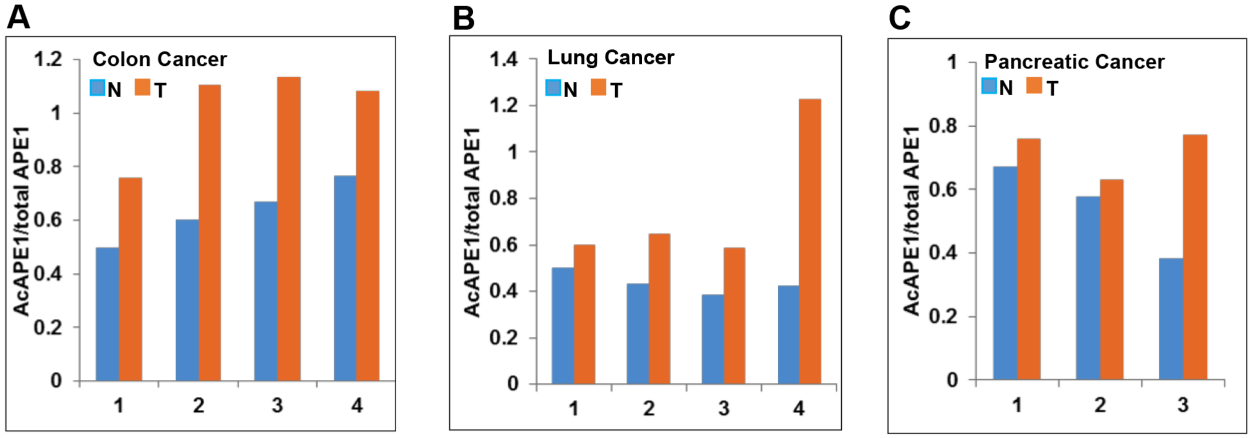

Supplementary Figure S1: The intensity of bands in Figure 1A-1C was quantitated (as integrated density, arbitrary unit) using Image J analysis tool. Histogram represents the relative amount of AcAPE1 in total APE1 in **A.** colon, **B.** lung and **C.** pancreatic cancer patients' non-tumor (N) and tumor (T) tissues.

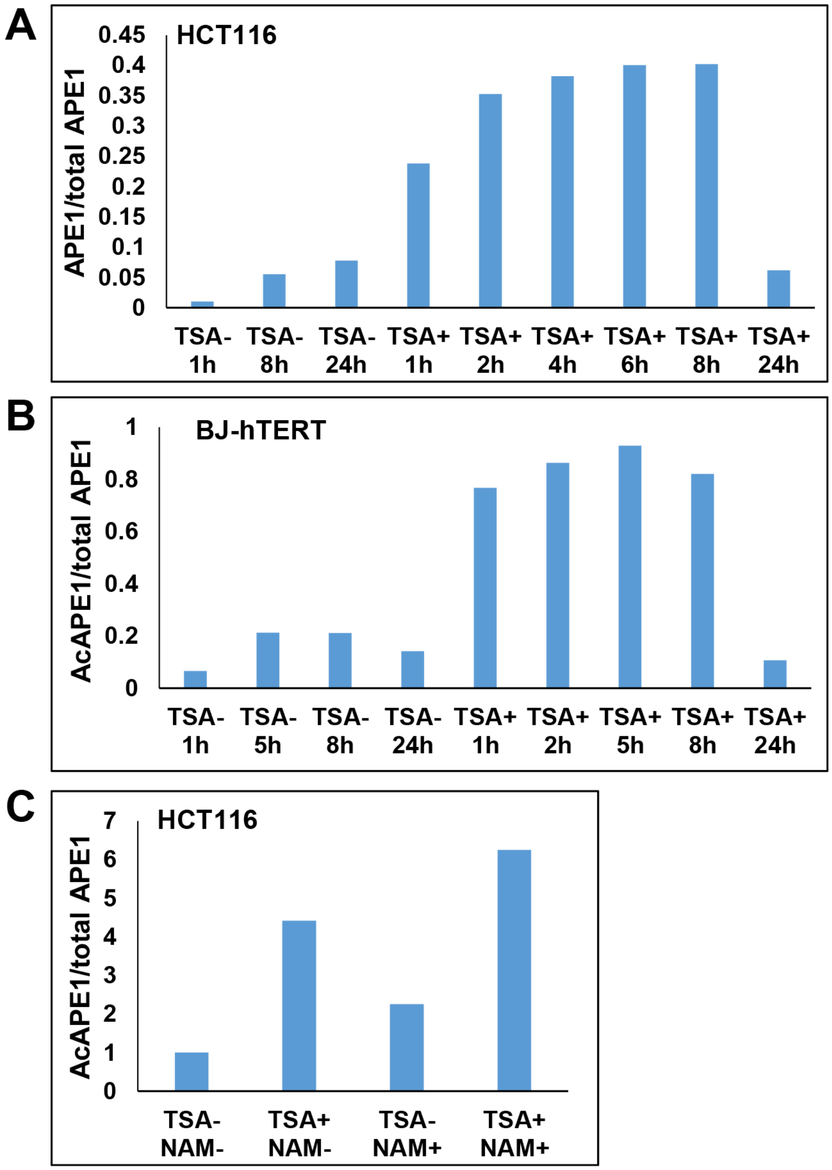

Supplementary Figure S2: Histogram shows the relative amount of AcAPE1 in total APE1 in TSA, NAM treated samples in Figure 2A-2C.

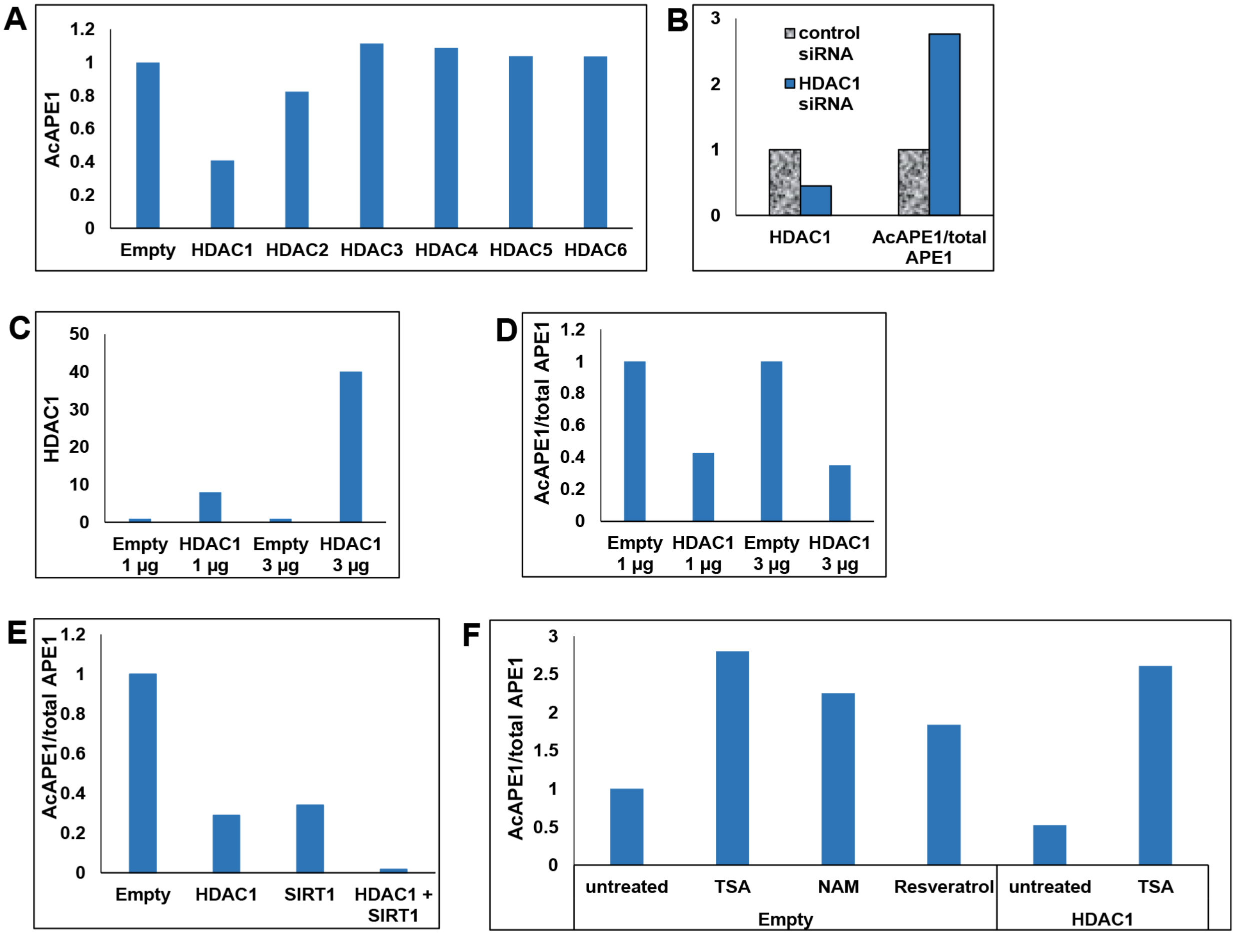

Supplementary Figure S3: Histogram shows the relative quantitation (as integrated density, arbitrary unit) of A. AcAPE1 in FLAG HDACs immunoprecipitates in Figure 3B, **B.** HDAC1 and AcAPE1/total APE1 in Figure 3C, **C.** HDAC1 in Figure 3D and **D-F.** AcAPE1/total APE1 in Figure 3D-3F.

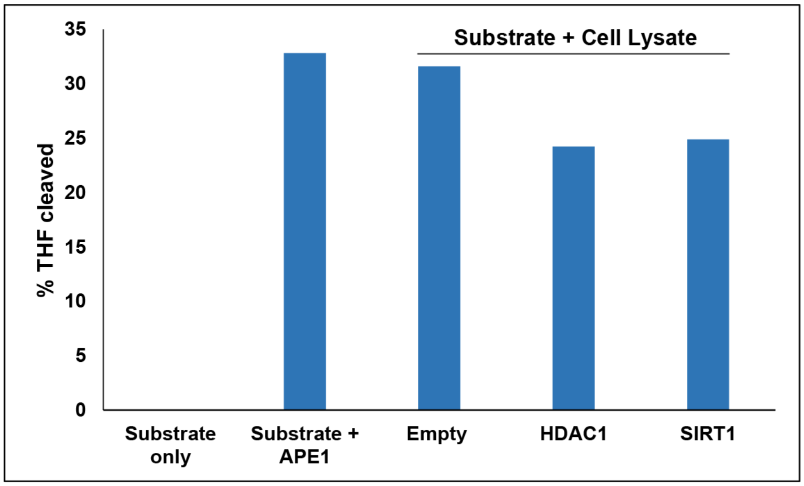

Supplementary Figure S4: Histogram shows the relative densitometric quantification of the percentage of cleaved product from the substrate in Figure 4B.

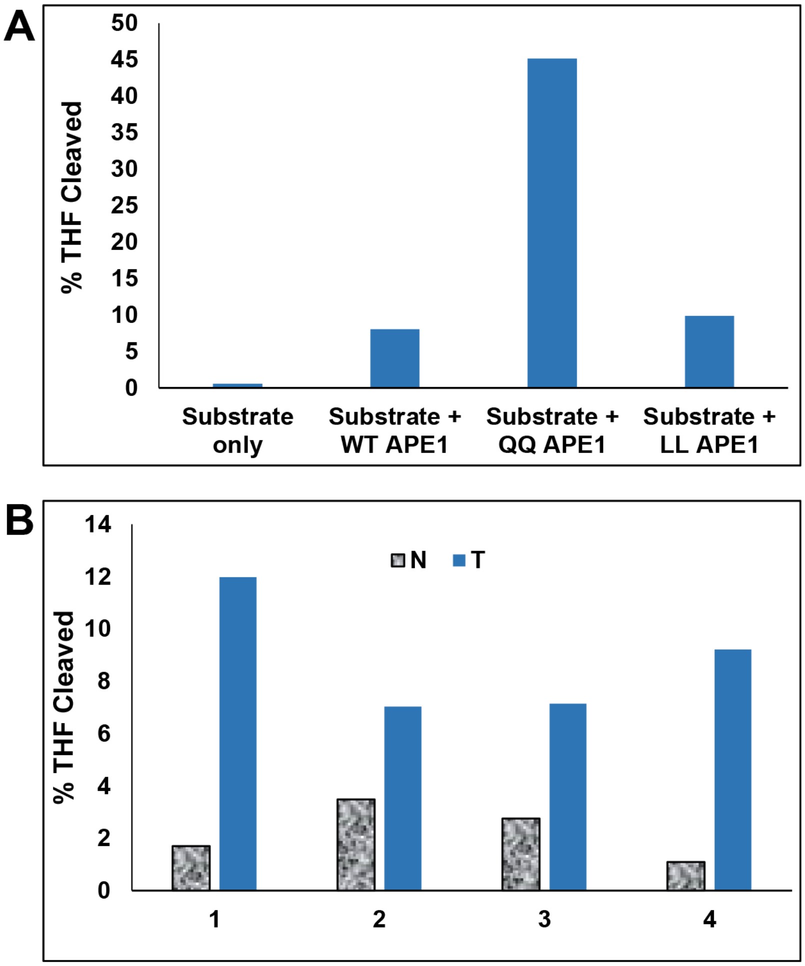

Supplementary Figure S5: Histogram shows the relative quantitation of the percentage of cleaved product from the substrate by A. WT vs. mutant APE1 proteins in Figure 5B and **B.** non-tumor (N) vs. tumor (T) tissue extracts from lung cancer patients in Figure 5C.

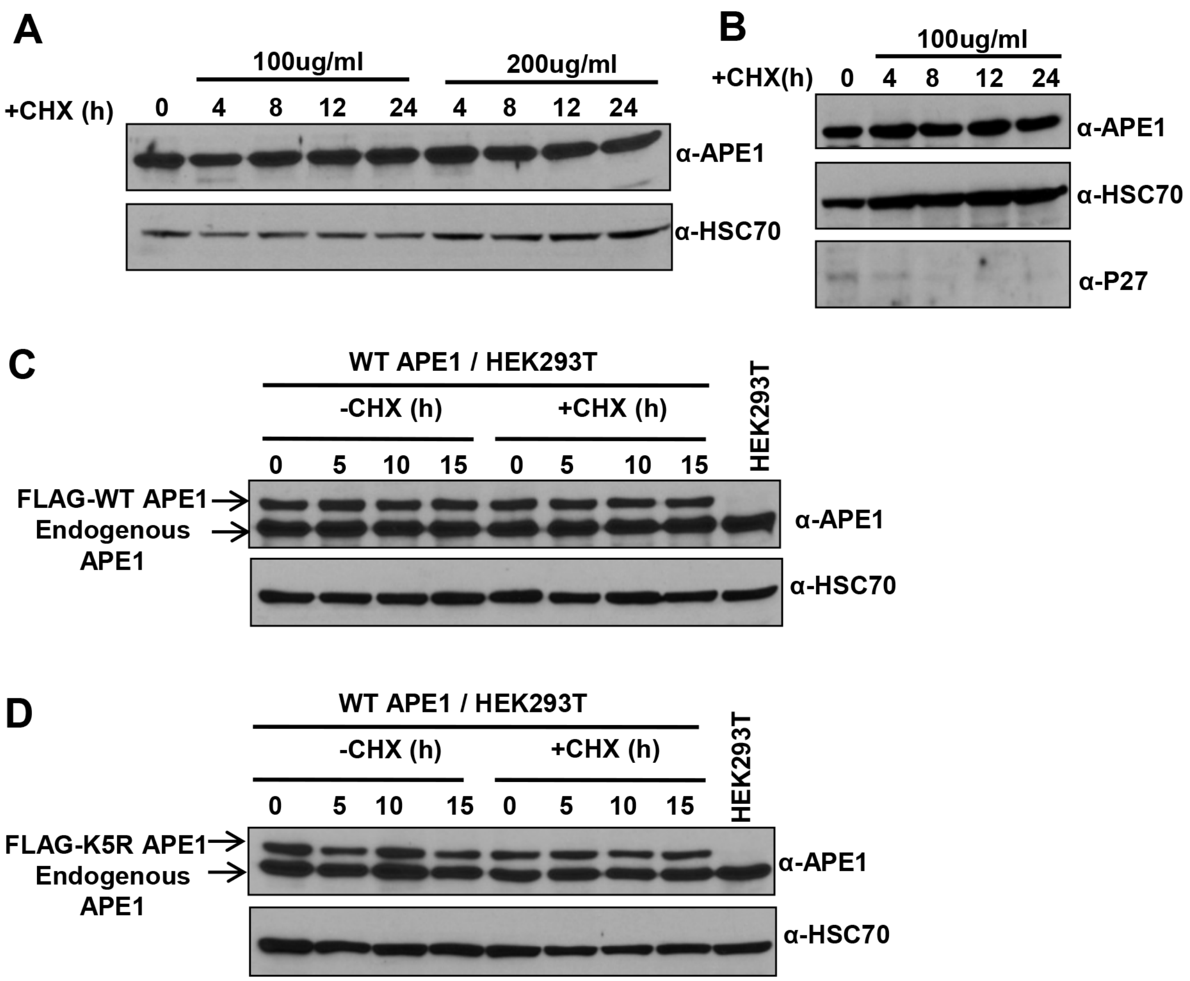

Supplementary Figure S6: A & B. Western blot analysis for endogenous APE1, p27 and HSC70 levels in cycloheximide (CHX) treated HEK293T cells. **C & D.** Western blot analysis for ectopic FLAG-tagged (C) WT and (D) K5R mutant APE1, endogenous APE1 and HSC70 levels in HEK293T cells transiently expressing FLAG-tagged WT or mutant APE1 proteins after treatment with CHX. p27 served as a positive control for CHX-mediated protein stability assay and HSC70 level used as a loading control.
